# Priming of Social Distance? Failure to Replicate Effects on Social and Food Judgments

**DOI:** 10.1371/journal.pone.0042510

**Published:** 2012-08-29

**Authors:** Harold Pashler, Noriko Coburn, Christine R. Harris

**Affiliations:** University of California San Diego, La Jolla, California, United States of America; Kyushu University, Japan

## Abstract

Williams and Bargh (2008) reported an experiment in which participants were simply asked to plot a single pair of points on a piece of graph paper, with the coordinates provided by the experimenter specifying a pair of points that lay at one of three different distances (close, intermediate, or far, relative to the range available on the graph paper). The participants who had graphed a more distant pair reported themselves as being significantly less close to members of their own family than did those who had plotted a more closely-situated pair. In another experiment, people's estimates of the caloric content of different foods were reportedly altered by the same type of spatial distance priming. Direct replications of both results were attempted, with precautions to ensure that the experimenter did not know what condition the participant was assigned to. The results showed no hint of the priming effects reported by Williams and Bargh (2008).

## Introduction

The term ‘priming’ has come to refer to a broad range of different behavioral effects that can be elicited by giving people a relatively incidental or minimal exposure to some words, pictures, or other stimuli, and then measuring some changes in behavior. Some priming effects are unquestionably robust. For example, inducing people to read a prime word (e.g., ‘doctor’) causes them to respond more quickly to a semantically associated target word presented shortly afterwards for a lexical decision response (e.g., pressing a “YES” button to verify that ‘nurse’ is indeed an English word) [1]. It also makes people more accurate in naming an associated target word when that word is briefly flashed and followed by a mask [2]. Perceptual priming effects of this sort have been directly replicated in hundreds of labs, and many of the published papers in this area have impressive statistical power, chiefly because they use experimental designs that feature within-subject comparisons with a large number of repeated measures for each participant for each of the experimental conditions.

### Perceptual versus Social/Goal Priming

These perceptual priming effects also appear straightforward from both mechanistic and functional perspectives. Studies using signal detection theory methods have shown that the effects reflect a perceptual bias toward interpreting ambiguous information about the target in line with the prime, rather than greater precision in the processing of prime-related inputs ([2]–[4]; see Pashler for an overview [5]). This sort of biasing process is naturally interpreted within a Bayesian framework as a rational short-term adaptation to the statistics governing the appearance of different objects in the world (see Huber, Shiffrin, Quach, and Lyle, for discussion [6]). The bias effect is often assumed to be implemented by a simple mechanism whereby spreading activation from the prime raises the baseline activation level of detector units corresponding to associated target items, which in turn thus require less stimulus input to reach threshold [7].

In recent years, however, a much more diverse set of priming effects have been reported in the psychological literature. These effects involve changes in how well people perform complex tasks, changes in their higher-level judgments about all kinds of matters, and even changes in their choice of actions or styles of actions. Well-known examples include reports that reading elderly-related words makes people walk more slowly when they leave the lab [8], reading money-related words makes people volunteer and donate less [9], seeing American flags makes US participants more favorably disposed to politically conservative points of view 8 months later [10], and plotting two points close together on a piece of graph paper makes people judge things from a greater “psychological distance” [11].

In comparing these social or goal priming studies to studies of perceptual priming, several things stand out. One is that a diverse and complex range of mechanisms would seem to be implicated, rather than anything as simple as pre-activation of detector units. A second is that direct replication attempts seem to be rare in this literature (as some priming researchers have acknowledged; see, e.g., Yong [12]). A third observation is that of the few direct replication attempts that have been publicly reported so far, at least a few have failed to find any of these priming effects whatsoever ([12], [13]; see also the General Discussion below). Due to well-known publication bias favoring publication of positive results, these few reports could potentially be concealing a far more widespread set of failures (although that obviously should not be assumed to be the case). Fourth, whereas perceptual priming has natural functional utility in making perception more effective, as noted above, the functional gains achieved by these higher-level priming effects are, at the very least, less self-evident. Moreover, if the activation of concepts automatically influences people's selection of goals and actions, this might subject them to random influences and even potentially to make them prone to being manipulated by others.

### Effect Sizes of Priming Effects

A final point of comparison between social/goal priming and perceptual priming relates to effect sizes, as measured with Cohen's d (size of an effect scaled against variability in the measured variable across the study population.) An examination of a small subset of the social/goal priming literature suggests that large effect sizes in the range from .5 to 1.0 are quite typical (see Table 1). Thus, by the metric of Cohen's d, reported social/goal priming effects look to be quite powerful.

**Table 1:** Effect sizes (Cohen's d) for a small number of well-known social and goal priming effects reported in the literature.

Social/Goal Priming Effect	Reference	Cohen's d
Priming with money made people work longer without requesting help	Vohs, Mead, and Goode (2006)	0.86
Priming with money made people volunteer to code fewer data sheets	Vohs, Mead, and Goode (2006)	0.66
Priming with money made people donate less money to the student fund	Vohs, Mead, and Goode (2006)	0.64
Priming with money made people place two empty chairs further apart	Vohs, Mead, and Goode (2006)	0.85
Seeing a flag makes people evaluate President Obama less favorably 8 months later	Carter, Ferguson, & Hassin (2011)	0.44
Reading elderly-related words slows people walking out of the lab.	Bargh, Chen, & Burrows (1996)	1.06
Plotting distant points on graph paper makes people report being more distant from their family and friends	Williams & Bargh^1^ (2008)	0.76

Footnotes:

1 Calculated based on the t value provided by the authors (−2.86) on the assumption of equal numbers of subjects in all 3 groups (28 per group.).

What about perceptual priming? Most perceptual priming studies have used within-subject designs and have not reported the variability of outcome measures across participants, making a calculation of Cohen's d values from published studies less than straightforward. To shed a bit of light on this, we chose a recent perceptual priming study that featured a rather typical design for a study of its type, exceptional statistical power, and seemed particularly carefully executed and analyzed ([14]; Experiment 1.) The study looked at how prime words affected reaction times to make lexical decisions for words that were either related or unrelated to the prime. The authors ran 80 participants, each of whom provided 80 data points in each of the conditions (6400 data points per cell). Using data obtained from the authors, we calculated that for the priming manipulation, Cohen's d was 0.06–generally considered a very small effect size. (The article reports a within-subject effect size measure, which is, naturally, larger; to calculate Cohen's d, we obtained additional numbers from the authors of the study.) In short, while the primes in this study produced results that were clearly statistically significant (p<.001), they actually accounted for only a tiny amount of the variability in participants' reaction times. Whether this is typical of perceptual priming effects is not known.

However, it does seem odd that social/goal priming studies should be dramatically *larger* than any reliable estimate of such a basic form of perceptual priming. As noted above, the presumed mechanism underlying perceptual priming is fairly direct: spreading activation pre-activates detector units, requiring less perceptual input to reach threshold [7]. The typical social or goal priming study, on the other hand, would seem to rely upon all of the same causal links as perceptual priming, but also a number of additional ones. For example, if reading words related to the elderly makes people walk more slowly [8], the effect would seem to rely not only upon reading the words and activating the concept of elderly people, but also retrieving an association from that to the concept of slow walking, modulating the participant's goals in line with the retrieved information, and implementing that modulation. Each of these additional steps involves processes that would be subject to many other competing influences, some being stable individual differences and some being very transitory influences. Each of these additional steps should have been expected to attenuate the effect size.

Thus, to find effect sizes in the social/goal priming literature that appear much larger than, or even just as big as, any in the perceptual priming literature strikes the current authors as rather curious. (A more definitive conclusion on this issue would require a meta-analysis that systematically analyzed raw datasets from the perceptual priming literature.)

The points described above certainly do not make a conclusive case either way about the validity of social/goal priming–they merely point out reasons why such results might deserve more scrutiny than they have thus far received. These points led us to believe that it would be useful to conduct an extensive series of direct replications of studies in the social/goal priming literature (a goal that several other labs have begun pursuing as well; [15]). The current experiment represents our attempt to replicate one well-known set of priming studies; other reports will be forthcoming from our lab.

The experiments we sought to replicate in the current study were reported by Williams and Bargh (2008). These authors described a new priming effect that, according to the authors, induces people to view things as lying at either a great or a small psychological distance from themselves. This was manifested in effects on several different kinds of judgments. Participants were asked to mark off a pair of points on a Cartesian plane, arranged so that the two dots were separated by a short, medium, or large distance relative to the entire grid. These authors reported that the closeness of the two dots that a participant plotted affected the participant's subsequent report of how emotionally close he or she felt to family members and friends. When the points were closer, people reported a greater degree of closeness. Williams and Bargh also reported that this “spatial priming” had a complex effect on participants' ratings of the estimated caloric content of different foods, with distance priming reducing caloric estimates for unhealthy, but not healthy foods.

The Williams and Bargh results have been cited 91 times (according to Google Scholar on June 9, 2012). As mentioned above, the substantive focus of their paper related to the concept of *psychological distance*. This familiar concept has been the subject of much research within the field of social cognition [16]. According to Trope and Liberman's *Construal Level Theory* [17], people view more distant events in a more abstract manner, and nearby events in a more concrete fashion. This prediction has been tested in studies that have, for example, asked people to imagine events that are temporally or spatially nearby or remote, and observing how they conceptualize these events (e.g., Fujita et al. [18]). In our view, the merits of Trope and Liberman's ideas about psychological distance are quite independent of whether plotting a few points on graph paper produces a “spillover” effect to influence the psychological distance with which a person views something else that they are exposed to soon after performing the graphing task.

## Study 1

The first replication attempt focused on Study 4 of Williams and Bargh (2008), in which the “spatial priming manipulation” was reported to have influenced people's feelings of emotional attachment to familiar people and places. According to Williams and Bargh (2008), participants who were primed by plotting close-together points on a piece of graph paper reported having stronger bonds to their parents, siblings, and hometown, as compared to those who had graphed distant points. The Williams and Bargh article did not mention specific precautions to prevent the experimenter(s) from knowing what condition the subjects were in (although it did report the use of a cover story; this issue is discussed further in the General Discussion below.) There were 84 participants in the Williams and Bargh study, and we tested a slightly greater number. The original study included an Intermediate priming condition as well as a Distant and Near condition; to maintain comparability, all three priming conditions were included, although the key findings of the original study involved the difference between the Distant and Near conditions.

### Method

#### Participants and Design

Ninety-two undergraduates (56 female, 36 male) at the University of California, San Diego participated individually in an experiment in return for partial fulfillment of a course requirement. The participants provided written informed consent. This research was approved by the University of California San Diego Social and Behavorial Sciences Institutional Review Board. Participants were randomly assigned to a condition.

#### Materials

The priming manipulation involved placing three points on an x,y coordinate plane. The current study had participants plot the same coordinates as Williams and Bargh (2008). The close distance pair was (2, 4) and (−3, −1), the intermediate distance pair was (8, 3) and (−6, −5), and the long distance pair was (12, 10) and (−11, −8). The total ranges on the x- and y-axes were 28 and 20, respectively. The close and long distance pairs were separated by 4 and 17 cm, respectively.

#### Procedure

The experimenter greeted participants and had them turn off their cellphones and complete a consent form while seated in the waiting room. They were then escorted individually into a private room to read task instructions. Following Williams and Bargh (2008), participants were given a cover story, namely that they had been recruited in order to provide feedback on some material to be used in a new type of standardized test. They were then provided with grid-style graph paper. Williams and Bargh (2008) provided the instructions, coordinates, and bond ratings together in a paper packet. To avoid experimenter expectancy effects, in the current experiment the plotting was done with paper and pencil but the instructions and ratings were communicated via the computer. The computer program presented the priming task instructions to subjects, and collected the ratings of bonds while the experimenter was in another room. Participants were instructed that once the experimenter had left the room, they should push the start button on the computer screen. The computer displayed more specific instructions informing participants that their task would be to plot two points on the graph paper in front of them, and providing them with the coordinates of the two points they should plot. Participants were also instructed by the computer to place this paper in a tray sitting to the left of the keyboard once they had completed the plotting task, and to press the space bar to continue. (It was not stated in the Williams and Bargh (2008) article whether the order of the dependent variables was randomized for each participant.) In the current study, the list of the three categories (parents, siblings, hometown) were presented in a different randomized order for each participant. On the next screen, participants were instructed to rate the strength of their bond to their parents, siblings, and hometown on a scale from 1 (not at all strong) to 7 (extremely strong). Once participants selected a bond rating for all three items presented, the SUBMIT button appeared at the bottom of the screen for participants to click.

After participants completed their emotional closeness ratings, the program presented a debriefing questionnaire that probed for suspicions about the relationship between the tasks. Participants were asked if they felt that any of the tasks were related and if so, to provide more detail as to exactly how. They were also asked if they experienced any issues plotting the coordinates, or rating the strength of their bonds, and to provide details if so. Finally, they were invited to provide comments or suggestions that might help in understanding their reaction to the experiment. When participants finished inputting their feedback, the program provided a full debrief on the computer screen, and then instructed participants to come out of the room to let the experimenter know they were finished.

### Results

In the debriefing questionnaire, one participant indicated suspicion regarding the purpose of the study. (This participant wrote “Yes, because it asked me to plot two point on a coordinate plane that were at opposite ends and far away from each other. Then it was followed by a series of questions involving strength of my bonds to certain people and places. I think that it has to do with perception.”). Six participants experienced problems plotting the points (e.g., plotting the wrong coordinates). These seven were excluded from the main analyses reported here; however, the pattern of results was not materially affected by whether these participants were included or excluded. In addition to the 7 participants above, another 14 participants reported on the debrief questionnaire that they felt that the ratings they were asked to provide did not fully pertain to their situation, making it hard to provide meaningful responses (e.g., they were an only child, their parents were deceased or estranged, etc.) The outcome of the statistical tests described below is not affected by the inclusion vs. exclusion of any of these subjects. Williams and Bargh (2008) do not indicate whether any of the subjects in the original experiment had difficulties in plotting or responding to questions.

After the 21 subjects were excluded, there were 71 subjects remaining (22 subjects in the close condition, 27 in the intermediate distance condition, and 22 in the far distance condition).

**Figure 1:**
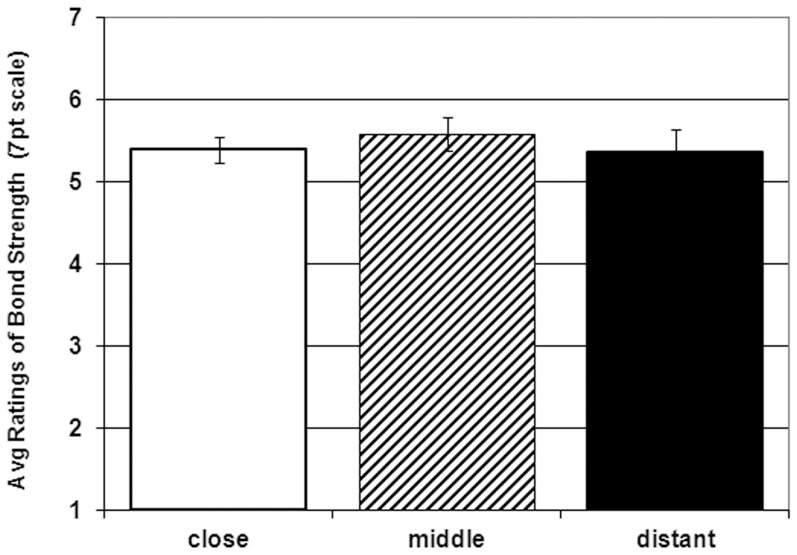
Participants' mean ratings of their closeness to their family and hometown as a function of what condition the subject was assigned to (error bars show standard error of the mean.).

A one-way ANOVA was used to test for differences in reported bond strength between the three spatial-prime groups. The ANOVA disclosed no significant difference, *F* (2, 68) = .31, *p* = .73). See Figure 1. There did not appear to be any trends of interest (mean closeness ratings were 5.36, SD = 1.28, for the distant priming, 5.58, SD = 1.05, for intermediate, and 5.39, SD = .75, for the close prime.) As previously mentioned, this is the case whether or not subjects are excluded for any of the reasons described above.

## Study 2

The second study attempted to replicate Study 3 of the same paper by Williams and Bargh (2008), which reported that physical-distance cues influenced participants' estimates of the caloric content of some “unhealthy foods” such as french fries and ice cream, and some “healthy foods” such as brown rice and apple. The rationale presented by Williams and Bargh for expecting an effect here was that “in our view, because caloric content is an affect-laden, potentially dangerous feature of unhealthy food, we hypothesized that people primed with distance would estimate that unhealthy food contains fewer calories, compared with people primed with closeness” (p. 305), with the intermediate-primed condition in between. (Although a creative hypothesis, it appears to us that the prediction that closeness primes will lead to people reporting that unhealthy foods, but not healthy foods, had fewer calories was not directly derivable from any theoretical ideas that were clearly articulated in the paper.) As in the first study, our goal was to perform as exact a replication as was feasible to do, while ensuring that the experimenter would remain blind to condition and have no possible opportunity to bias the results. There were 59 participants in the original study.

### Method

#### Participants and Design

Ninety-two undergraduates (58 female, 34 male) participated for partial fulfillment toward course credit. Subjects were randomly assigned to one of the same three priming conditions described in the first experiment. The participants provided written informed consent, and the research was approved by the University of California San Diego Social and Behavorial Sciences Institutional Review Board.

#### Materials

The priming materials were the same as Experiment 1.

#### Procedures

The procedures were the same as in Experiment 1, except that instead of rating bonds to family and hometown, participants were shown via computer a list of 10 food items and asked to estimate the calories in a single serving of each, and to enter that number next to the item. Following Williams and Bargh, 5 items commonly viewed as “healthy foods” (yogurt, oatmeal, brown rice, apple, baked potato) were listed, along with 5 “unhealthy foods” (ice cream, french fries, potato chips, chocolate bar, cheeseburger). The 10 items on the list were presented in a different random order for each participant. (It is unclear from the methods section of Williams and Bargh (2008) whether the same was done in their study, but this appeared to us to be the most appropriate way of implementing the basic design.) Once participants typed in a value for every item on the list, the SUBMIT button appeared at the bottom of the screen for them to click on. The same debrief questionnaire used in the previous study was employed here as well, except that instead of a question pertaining to bond ratings, participants were asked if they had difficulty estimating the calories for the food items presented to them and what strategy (if any) they used for this task.

### Results

Four participants were removed from the final analyses because they experienced problems plotting the coordinates (e.g., coloring in the entire square rather than plotting points). No participant indicated clear suspicion of the priming manipulation or purpose of the study. The exclusion of the four left 28 in the close condition, 31 in the intermediate distance condition, and 29 in the far distance condition. (The Williams and Bargh article does not mention whether any subjects were excluded from this experiment for any reason.)

**Figure 2:**
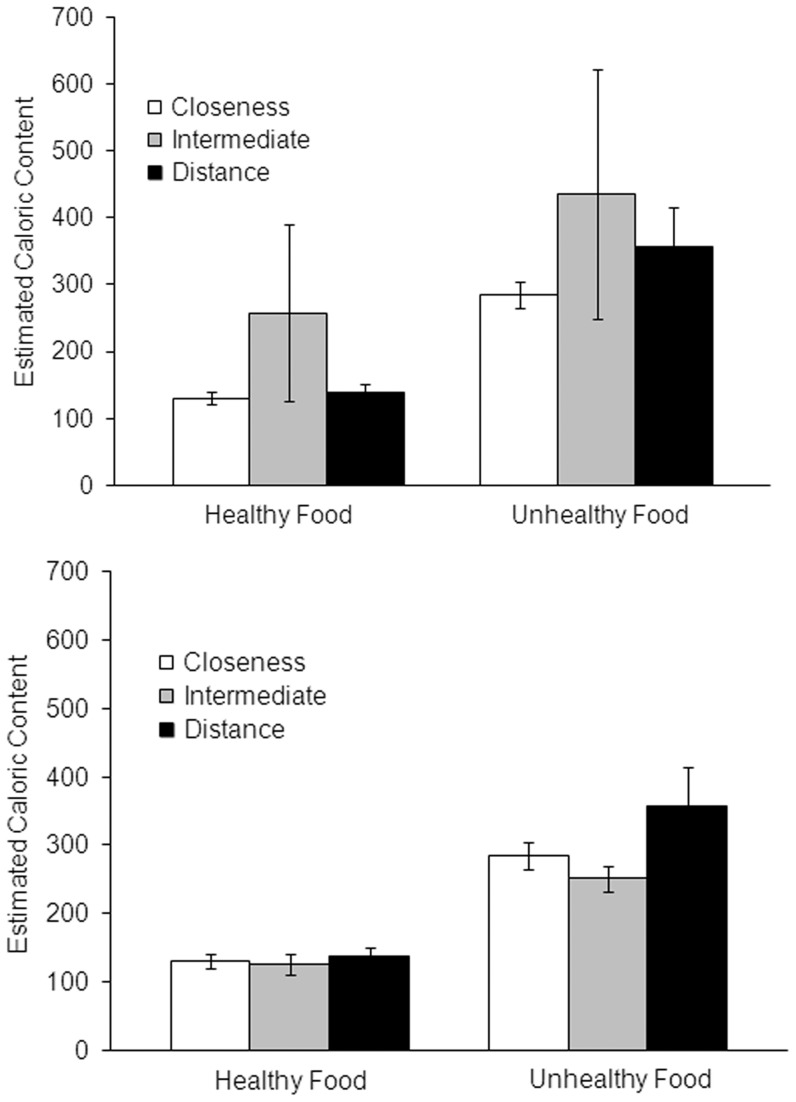
Mean estimates of the calorie content of some foods as a function of what priming condition the subject was assigned to and whether the food was from a list of healthy foods or unhealthy foods (error bars show standard error of the mean). Top panel: all subjects; Bottom panel: with data removed from one outlier subject in the Intermediate group who gave estimates approximately one order of magnitude in excess of the average (see text).

**Figure 3:**
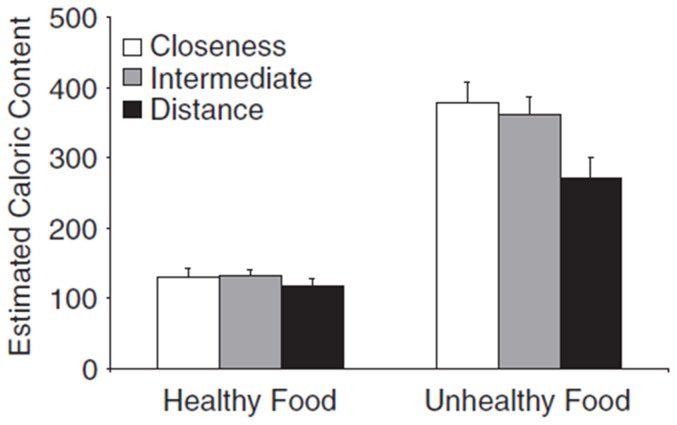
Results of Williams and Bargh (2008).

Each participant's mean calorie estimates were analyzed with a 3×2 mixed design ANOVA with spatial priming (close, intermediate, distant) as a between subjects factor and food type (healthy vs. unhealthy) as a within-subjects factor. Figure 2 shows the mean estimates for healthy and unhealthy food groups across the three priming conditions. The results did not show any hint of the effects reported by Williams and Bargh (2008) (Figure 3 displays the results of Williams and Bargh, 2008, for comparison.) For unhealthy foods, the close spatial priming group actually yielded lower calorie estimates (M = 284.7, SD = 101.2) compared to the distant priming group (M = 357.6, SD = 311.9), although not significantly so.

There was no main effect of priming condition, *F* (2,85) = .53, p = .59. Williams and Bargh (2008) found a significant interaction between priming condition and healthy vs. unhealthy foods. Our results showed no significant interaction, *F* (2, 85) = .45, p = .64. The top panel of Figure 2 shows higher mean calorie estimates for subjects in the Intermediate priming condition relative to the other two priming conditions. This was due to the effect of one subject who estimated that unhealthy and healthy foods had 6000 and 4200 calories (per serving size), respectively–estimates approximately one order of magnitude greater than those given by the typical subject. On the debrief questionnaire, this participant reported having problems with the Cartesian grid because it was not labeled. When the ANOVA was re-run with this subject removed, the results showed no significant effect of priming condition, as seen in the lower panel of Figure 2, *F* (2, 84) = 2.10, p = .13. In fact, the data show a nonsignificant trend in the opposite direction from the effect reported by Williams and Bargh (2008). There was also no significant interaction in this analysis, *F* (2, 84) = 1.88, p = .16. (This remained true even if the four subjects excluded for plotting problems were retained.)

As another way to assess the central tendency of the data with the effect of outliers minimized, we computed median ratings for each particular food from the ratings given by the subjects in each of the three conditions, and then averaged these medians across all 5 different foods in the healthy and unhealthy categories (see Table 2). For both healthy and unhealthy food, the median calorie estimates show little in the way of differences–but the medians are again highest for the Distant primed group, rather than lowest as reported by Williams and Bargh. To sum up, the pattern of results reported here offers no support whatsoever for the findings of Williams and Bargh (2008), and it seems fair to say, no reason to believe that the primes had any effect upon food calorie estimation.

**Table 2:** Mean calorie estimates (computed across items) of the median ratings (computed across the subjects assigned to each condition) for the unhealthy and healthy foods.

	Food Type	
Priming Condition	Unhealthy	Healthy
Closeness	250	114
Intermediate	254	102
Distance	264	122

## Discussion

The present work attempted two fairly exact replications of studies reported by Williams and Bargh (2008). Neither study supported the original results. This naturally raises the question: why are these two studies yielding discrepant outcomes?

### Possible Explanations for the Discrepancy

Although the Williams and Bargh paper did not discuss this issue, we had initially supposed that experimenter expectancy effects might have played a role in producing the different outcomes of the two studies. However, we have recently learned from the first author of that paper that precautions against experimenter expectancy effects were indeed present in the original study. L.E. Williams (personal communication, April 12, 2012) stated that “…experimenters [were kept] blind to condition” and that this was accomplished as follows: “the … materials were presented in a stapled paper packet; the prime was never the top page in the packet…” and “the study materials were handed out in a random order, and the studies were not completed in the immediate presence of the experimenter (e.g., the experimenter sat outside of a dining hall, students took the study with them into the dining hall and returned afterward or the experimenter sat outside, passerby took the study with them on a clipboard to nearby stairs/seating, and later returned the clipboard).” Thus, it appears that possible experimenter expectancy effects in the original study are not likely to be accounting for the difference in outcomes.

A second, seemingly minor difference between the replication attempt and the original relates to the fact that the instructions were provided by computer in our studies, whereas they were provided in writing in the Williams and Bargh paper. Logically, this could be the reason for the discrepancy. However, we tend to doubt it in light of very recent developments. Since completing our studies, we have learned of another independent attempt to directly replicate the Williams and Bargh (2008) Study 4, carried out as part of a well-publicized effort recently initiated to estimate the overall replicability rate for results published in *Psychological Science* and certain other behavioral science journals [19]. R. Clay (personal communication, May 23, 2012) reports running 124 subjects and finding neither significant results nor any trends in line with the original Williams and Bargh results (all Fs<1). In the Clay study, the instructions were provided in writing (as in the original study) but the experimenter remained present in order to answer any possible questions.

Thus, there are now two failures to replicate Williams and Bargh (2008, Study 4) using both computerized instructions with the experimenter absent, and also written instructions with the experimenter present. The original study apparently involved written instructions with the experimenter absent. While one cannot logically rule out the idea that these two seemingly minor features dramatically and perversely interact in determining whether priming occurs, this seems to us to be rather far-fetched. It should also be acknowledged, however, that there could be other unsuspected differences that somehow affect the observations, such as differences in the participants (although all the studies mentioned involved college students and it is not clear to us what such differences might be.) We would also note that the literature contains one published study [20], which used the same distance priming manipulation and found effects on an unrelated measure (ratings of amusement at the description of an unusual sexual practice), which perhaps weighs slightly in favor of the idea that these kinds of primes can have some effects.

Another possibility, however, is that the Williams and Bargh results are simply not valid, representing, for example Type 1 errors. The likely rate of such errors in this literature is a point of controversy and, at this point, speculation [15], [21]. Due to the “file drawer problem” (the notorious tendency for scientific journals to selectively publish positive outcomes; cf Rosenthal [22]; see also Simmons, Nelson and Simonsohn [23]) the published literature could potentially provide a very inaccurate picture of the totality of the data that have been collected. This uncertainty likely reflects the fairly widespread disinclination of typical journals (although, fortunately, not *PLoS One*) to publish failures to replicate (a disinclination that is increasingly recognized as injurious to the credibility of many scientific fields [24], [25], [26]).
